# Antibiotic resistance in Escherichia coli from Nigerian students, 1986 1998.

**DOI:** 10.3201/eid0604.009913

**Published:** 2000

**Authors:** I N Okeke, S T Fayinka, A Lamikanra

**Affiliations:** Obafemi Awolowo University, Ile-Ife, Nigeria.

## Abstract

We tested 758 fecal Escherichia coli isolates, recovered from Nigerian students in 1986, 1988, 1990, 1994, and 1998, for susceptibility to seven antimicrobial drugs. The prevalences of strains resistant to tetracycline, ampicillin, chloramphenicol, and streptomycin were 9% to 35% in 1986 and 56% to 100% in 1998. These findings demonstrate that resistance gene reservoirs are increasing in healthy persons.


### Antibiotic Resistance in *Escherichia coli* from Nigerian Students, 1986–1998

**Iruka N. Okeke, Susan T. Fayinka, and Adebayo Lamikanra**
Obafemi Awolowo University, Ile-Ife, Nigeria

We tested 758 fecal *Escherichia coli* isolates, recovered from Nigerian students in 1986, 1988, 1990, 1994, and 1998, for susceptibility to seven antimicrobial drugs. The prevalences of strains resistant to tetracycline, ampicillin, chloramphenicol, and streptomycin were 9% to 35% in 1986 and 56% to 100% in 1998. These findings demonstrate that resistance gene reservoirs are increasing in healthy persons.

Resistance to antibiotics is highly prevalent in bacterial isolates worldwide, particularly in developing countries (1-4). Routine monitoring of antibiotic resistance provides data for antibiotic therapy and resistance control (5). Normal intestinal flora are a reservoir for resistance genes; the prevalence of resistance in commensal *Escherichia coli* is a useful indicator of antibiotic resistance in bacteria in the community (6,7). Studies with *E. coli* are of particular relevance because this species can occupy multiple niches, including human and animal hosts (8). In addition, *E. coli* strains efficiently exchange genetic material with pathogens such as *Salmonella, Shigella, Yersinia*, and *Vibrio* species, as well as pathogenic *E. coli* (8,9).

Few studies have evaluated antimicrobial resistance in sub-Saharan Africa. Most available data are specific to pathogenic organisms, and trends over time in this region are rarely followed. We monitored trends in antibiotic resistance prevalence in *E. coli* isolates from apparently healthy Nigerian students by measuring resistance to seven antimicrobial drugs in *E. coli* isolated from 758 stool specimens collected over a 13-year period.

### The Study

Stool specimens were collected from apparently healthy student volunteers at the Obafemi Awolowo University in 1986, 1988, 1994, 1996, and 1998. The students, who provided informed consent, were 16 to 32 years of age; 347 (45.8%) were female. All volunteers who had taken antimicrobial drugs or been ill in the previous month were excluded from participation.

The specimens were collected into Stuart's transport medium and subcultured onto MacConkey agar plates. Colonies with morphologic characteristics of *E. coli* were subcultured onto fresh plates. Identity was confirmed by conventional biochemical tests. The standard disk diffusion method was used for susceptibility testing (10). The antibiotic disks used were ampicillin (10 µg), chloramphenicol (30 µg), streptomycin (30 µg), sulfisomidine (250 µg), nalidixic acid (30 µg), trimethoprim (5 µg), and tetracycline (30 µg) (AB Biodisk, Sweden). *E. coli* NCTC 10418 and K-12 C600 were used as controls. All isolates recovered at the same time from the same person with identical biochemical and antibiotic susceptibility profiles were considered identical. The prevalence of resistance to each drug for each year was computed. Trends were analyzed by Pearson's regression and the chi-square test for trend (Mantel-Haenszel test).

No sampling was conducted in 1990 or 1992, and streptomycin resistance was not tested in 1994. Despite these gaps in the study, trends toward increasing resistance in *E. coli* were observed with tetracycline, sulfonamides, ampicillin, chloramphenicol, and streptomycin (Figure). The proportion of isolates resistant to chloramphenicol increased from 13.5% in 1986 to 59.8% in 1998, while isolates resistant to tetracycline increased from 34.9% to 100%. The trends for tetracycline and streptomycin were statistically significant ($p < 0.05$, Pearson's

Address for correspondence: A. Lamikanra, Department of Pharmaceutics, Faculty of Pharmacy, Obafemi Awolowo University, Ile-Ife, Nigeria; fax: 234-36-231-733; e-mail: alamikan@oauife.edu.ng.

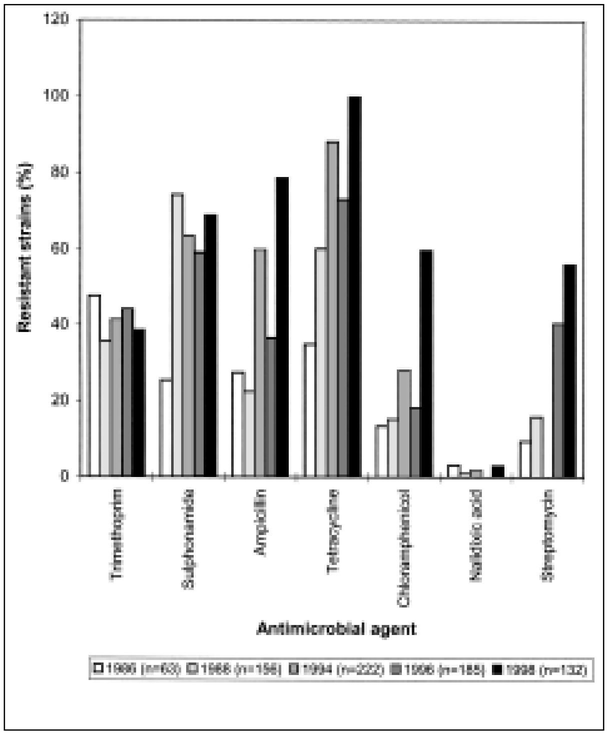

Figure. Percentage of isolates resistant to seven antimicrobial drugs, 1986-1998.
*No data are available for streptomycin in 1994.

Table. Proportions of universally susceptible and multiply resistant *Escherchia coli*, Nigeria, 1986–1998

| Yr/(No. isolates) | No. (%) isolates Sensitive to all drugs tested | Resistant to 3 drugs | Resistant to 6 drugs |
|---|---|---|---|
| 1986/(63) | 19 (30.2) | 19 (30.2) | 1 (1.6) |
| 1994/(222)[a] | 24 (15.4) | 37 (23.7) | 0 (0) |
| 1996/(185) | 34 (18.4) | 105 (56.8) | 6 (3.2) |
| 1998/(132) | 0 (0) | 93 (70.5) | 21 (15.9) |
| p value[b] | -0.768 (0.232) | 0.960 (0.04) | 0.768 (0.232) |

[a]Streptomycin was not tested in 1994.
[b]p value based on Pearson's regression coefficient for trend.

regression; p <0.10, chi-square test for trend). The prevalence of sulfonamide resistance increased sharply from 1986 to 1988 (from 25.4% to 74.3%), then remained fairly constant. The moderate levels of resistance to trimethoprim (35.7% and 47.6%) and low levels to nalidixic acid (0% to 3.2%) did not change appreciably.

As strains susceptible to all drugs became less common, the proportion of isolates resistant to multiple antibiotics increased (Table). In 1986, 19 (30.2%) isolates were susceptible to all the drugs tested, but by 1998 all the isolates were resistant to at least one drug. The proportion of isolates resistant to three or more drugs increased steadily, from 30.2% in 1986 to 70.5% in 1998 (p <0.05, Pearson's regression; p <0.10, chi-square test for trend). Only one isolate from 1986 was resistant to at least six drugs, but 15.9% of the isolates obtained in 1998 demonstrated this phenotype. Three isolates were resistant to all drugs tested. Two of these had only low levels of resistance to nalidixic acid and chloramphenicol (in both cases) and ampicillin in one case. One isolate recovered in 1998 had high-level resistance to all the drugs tested, as well as to cefalotin and fluoroquinolones (ofloxacin, ciprofloxacin, and norfloxacin); however, it was susceptible to spectinomycin, tobramycin, and gentamicin.

### Conclusions

Residents in and visitors to developing countries acquire antibiotic-resistant *E. coli* as part of their normal flora (1,2,11). Our data show that the prevalence of resistance to most drugs tested in *E. coli* isolates from apparently healthy students is within the high range reported previously (2) and has increased from 1986 to 1998. The increases in prevalence of resistance to streptomycin and tetracycline were statistically significant. In most drugs tested, the proportion of resistance isolates has increased rapidly, so that the usefulness of drugs moderately effective in 1986 has been severely compromised. The prevalence of resistance in these commensal *E. coli* during the latter sampling points reached >50% in 1998 for all drugs except trimethoprim and nalidixic acid. For tetracycline, the proportion of resistant strains increased from <40% to 100% in the 13-year period. As in other studies (2), the general trend toward increasing prevalence of resistance was marked by the recovery of an increasing proportion of strains that were simultaneously resistant to several drugs. These data sound a warning because the indiscriminate use of antibiotics, along with poor hygiene and infection control (risk factors for antibiotic resistance in bacteria) are highly prevalent in Nigeria and other developing countries (4,12).

The five drugs for which a considerable rise in resistance was seen from 1986 to 1998

(ampicillin, sulfonamides, streptomycin, chloramphenicol, and tetracycline) are extensively used in Nigeria and other developing countries (4,12). These five inexpensive drugs are widely available without prescription from authorized health institutions and pharmacies, as well as from unauthorized patent medicine shops and other distributors (4,12). Streptomycin, which must be injected, and chloramphenicol, because of its toxicity, are less popular than the other three drugs—and had lower resistance prevalence—but are nonetheless used more often than indicated. The prevalence of resistance to a sixth agent, nalidixic acid, remained low, corresponding to the low consumption of this and related drugs in health institutions and the community. The recent introduction of fluoroquinolones in clinical practice may alter this profile, since resistance to the fluoroquinolones and nalidixic acid has been shown to spread rapidly (3,13).

Resistance to trimethoprim was higher than to other drugs in 1986 but did not increase substantially in subsequent years. This drug is heavily used in health institutions and in the community, generally in combination with sulfamethoxazole. The selective pressure generated by overuse explains the relatively high prevalence of resistance in *E. coli* isolates in 1986. However, it is not clear why the trend observed with other widely used drugs was not seen in this case. Any increases in prevalence of strains resistant to trimethoprim may have occurred before 1986. A similar plateau with sulfonamide resistance followed a rapid rise from 1986 to 1988. Why this plateau was seen with two drugs and not others is not known, but the observation has been reported in other locations (14).

Ingestion of antibiotics is known to provide selective pressure ultimately leading to a higher prevalence of resistant bacteria, even among persons who have not taken antibiotics (7,8). As recent antibiotic use was a criterion for exclusion from the study, selection of the resistance strains isolated in the study may have occurred before the volunteer hosts were colonized. The source of resistant organisms in our study population is not known, but possible sources are food, water, and person-to-person transfer. Suboptimal sanitary conditions and overcrowding in student hostels may facilitate the spread of these organisms.

We observed rapid increases in the prevalence of resistance in commensal *E. coli* to most of the older, less expensive antimicrobial drugs used in the management of infections in Nigeria. Not only are these strains potential causes of infection, but they are also potential reservoirs of resistance genes that could be transferred to pathogens. For this reason, the trends seen with commensal *E. coli* may also occur with pathogenic organisms. Studies in other developing countries have shown that the trend in enteric pathogens is toward increasing antibiotic resistance (3). Our study emphasizes the need to monitor commensal organisms as well as pathogens by susceptibility testing to guide treatment. Control of antibiotic resistance is needed to conserve the usefulness of the remaining drugs. The data suggest that nalidixic acid and possibly trimethoprim may be useful in treating infections caused by pathogenic *E. coli* and other related bacteria in Nigeria. The future usefulness of these drugs will, however, depend on effective interventions to halt the selection and spread of resistance among enteric organisms.

### Acknowledgments

We thank Bukola Quadri, Oladipo Ojo, and Kemi Adeyemi for technical assistance.

This work was funded by grants from the International Program in the Chemical Sciences, Uppsala University, Sweden, and the Obafemi Awolowo University Research Council, Ile-Ife, Nigeria.

Dr. Okeke, a lecturer in the Department of Pharmaceutics, Obafemi Awolowo University, Ile-Ife, Nigeria, is a fellow in the Department of Microbiology and Immunology and the Center for Vaccine Development of the University of Maryland School of Medicine. Her research interests focus on epidemiologic and genetic studies of *E. coli*.

### References

1. Calva JJ, Sifuentes-Osornio J, Ceron C. Antimicrobial resistance in fecal flora: longitudinal community-based surveillance of children from urban Mexico. Antimicrob Agents Chemother 1996;40:1699-702.
2. Lamikanra A, Okeke IN. A study of the effect of the urban/rural divide on the incidence of antibiotic resistance in *Escherichia coli*. Biomed Lett 1997;55:91-7.
3. Hoge CW, Gambel JM, Srijan A, Pitarangsi C, Echeverria P. Trends in antibiotic resistance among diarrheal pathogens isolated in Thailand over 15 years. Clin Infect Dis 1998;26:341-5.
4. Hart CA, Kariuki S. Antimicrobial resistance in developing countries. Br Med J 1998;317:647-50.

5. O'Brien T. The global epidemic nature of antimicrobial resistance and the need to monitor and manage it locally. Clin Infect Dis 1997;24(Suppl 1):S2-8.
6. Levy S, Marshall B, Schleuderberg S, Rowe B, Davis J. High frequency of antibiotic resistance in human fecal flora. Antimicrob Agents Chemother 1988;32:1801-6.
7. Levin B, Lipsitch M, Perrot V, Schrag S, Antia R, Simonsen L, et al. The population genetics of antibiotic resistance. Clin Infect Dis 1997;24:S9-16.
8. Levy SB. Antibiotic resistance: an ecological imbalance. Ciba Found Symp 1997;207:1-9; discussion 9-14.
9. Tauxe RV, Cavanagh TR, Cohen ML. Interspecies transfer in vivo producing an outbreak of multiply resistant Shigellosis. J Infect Dis 1989;160:1067-70.
10. National Committee for Clinical Laboratory Standards. Methods for dilution antimicrobial susceptibility tests for bacteria that grow aerobically. 2nd ed. Approved standard. Villanova (PA): The Committee; 1990.
11. Murray BE, Mathewson JJ, DuPont HL, Ericsson CD, Reves RR. Emergence of resistant fecal *Escherichia coli* in travelers not taking prophylactic antimicrobial agents. Antimicrob Agents Chemother 1990;34:515-8.
12. Okeke IN, Lamikanra A, Edelman R. Socioeconomic and behavorial factors leading to acquired bacterial resistance to antibiotics in developing countries. Emerg Infect Dis 1999;5:18-27.
13. Green S, Tillotson G. Use of ciprofloxacin in developing countries. Pediatr Infect Dis J 1997;16:150-9; discussion 160-2.
14. O'Brien T. Resistance of bacteria to antibacterial agents: report of Task Force 2. Reviews of Infectious Diseases 1987;9:S244-60.
